# Systematic Review and Meta-Analysis of Tocilizumab Therapy versus Standard of Care in over 15,000 COVID-19 Pneumonia Patients during the First Eight Months of the Pandemic

**DOI:** 10.3390/ijerph18179149

**Published:** 2021-08-30

**Authors:** Naim Mahroum, Abdulla Watad, Charlie Bridgewood, Muhammad Mansour, Ahmad Nasr, Amr Hussein, Rola Khamisy-Farah, Raymond Farah, Omer Gendelman, Merav Lidar, Yehuda Shoenfeld, Howard Amital, Jude Dzevela Kong, Jianhong Wu, Nicola Luigi Bragazzi, Dennis McGonagle

**Affiliations:** 1 Zabludowicz Center for Autoimmune Diseases, Department of Medicine B., Sheba Medical Center, Ramat Gan 5265601, Israel; naim.mahroum@gmail.com (N.M.); watad.abdulla@gmail.com (A.W.); Omer.Gendelman@sheba.health.gov.il (O.G.); Yehuda.Shoenfeld@sheba.health.gov.il (Y.S.); Howard.Amital@sheba.health.gov.il (H.A.); 2 Sackler Faculty of Medicine, Tel Aviv University, Tel Aviv 6997801, Israel; Merav.Lidar@sheba.health.gov.il; 3 International School of Medicine, Istanbul Medipol University, Istanbul 34810, Beykoz, Turkey; 4 Leeds Institute of Rheumatic and Musculoskeletal Medicine (LIRMM), University of Leeds, Leeds LS7 4SA, UK; C.D.Bridgewood@leeds.ac.uk (C.B.); D.G.McGonagle@leeds.ac.uk (D.M.); 5 Rheumatology Unit, Sheba Medical Center, Ramat Gan 5265601, Israel; 6 Department of Surgery A, Galilee Medical Center, Nahariya 2210001, Israel; Hmode_220@hotmail.com; 7 Faculty of Medicine of the Galilee, Bar-Ilan University, Safed 13100, Israel; 8 Division of General Surgery, St. Michael’s Hospital, Unity Health Toronto, University of Toronto, Toronto, ON M5B 1W8, Canada; 9 Department of Pathology, Papa Giovanni XXIII Hospital, 24127 Bergamo, Italy; a.nasr@campus.unimib.it; 10 Department of Pathology, University of Milano-Bicocca, 20126 Milan, Italy; 11 Medical Faculty, University of Parma, 43125 Parma, Italy; amrhussein@hotmail.it; 12 Clalit Health Service, Akko, Azrieli Faculty of Medicine, Bar-Ilan University, Safed 13100, Israel; rkhamisy@yahoo.com; 13 Department of Internal Medicine, Ziv Medical Center, Safed 13100, Israel; raymond.f@ziv.health.gov.il; 14 Medical Department, Saint Petersburg State University, 199034 Saint Petersburg, Russia; 15 Ariel University, Kiryat HaMada 3, Ariel 40700, Israel; 16 Laboratory for Industrial and Applied Mathematics (LIAM), Department of Mathematics and Statistics, York University, Toronto, ON M3J 1P3, Canada; jdkong@yorku.ca (J.D.K.); wujhhida@gmail.com (J.W.); 17 Postgraduate School of Public Health, Department of Health Sciences (DISSAL), University of Genoa, 16132 Genoa, Italy; 18 Chapel Allerton Hospital, Chapeltown Road, Leeds LS7 4SA, UK

**Keywords:** COVID-19, tocilizumab, systematic review and meta-analysis

## Abstract

Background. Tocilizumab is an anti-IL-6 therapy widely adopted in the management of the so-called “cytokine storm” related to SARS-CoV-2 virus infection, but its effectiveness, use in relation to concomitant corticosteroid therapy and safety were unproven despite widespread use in numerous studies, mostly open label at the start of the pandemic. Methods: We performed a systematic review and meta-analysis of case-control studies utilising tocilizumab in COVID-19 on different databases (PubMed/MEDLINE/Scopus) and preprint servers (medRxiv and SSRN) from inception until 20 July 2020 (PROSPERO CRD42020195690). Subgroup analyses and meta-regressions were performed. The impact of tocilizumab and concomitant corticosteroid therapy or tocilizumab alone versus standard of care (SOC) on the death rate, need for mechanical ventilation, ICU admission and bacterial infections were assessed. Results. Thirty-nine studies with 15,531 patients (3657 cases versus 11,874 controls) were identified. Unadjusted estimates (*n* = 28) failed to demonstrate a protective effect of tocilizumab on survival (OR 0.74 ([95%CI 0.55–1.01], *p* = 0.057), mechanical ventilation prevention (OR 2.21 [95%CI 0.53–9.23], *p* = 0.277) or prevention of ICU admission (OR 3.79 [95%CI 0.38–37.34], *p* = 0.254). Considering studies with adjusted, estimated, tocilizumab use was associated with mortality rate reduction (HR 0.50 ([95%CI 0.38–0.64], *p* < 0.001) and prevention of ICU admission (OR 0.16 ([95%CI 0.06–0.43], *p* < 0.001). Tocilizumab with concomitant steroid use versus SOC was protective with an OR of 0.49 ([95%CI 0.36–0.65], *p* < 0.05) as was tocilizumab alone versus SOC with an OR of 0.59 ([95%CI 0.34–1.00], *p* < 0.001). Risk of infection increased (2.36 [95%CI 1.001–5.54], *p* = 0.050; based on unadjusted estimates). Conclusion: Despite the heterogeneity of included studies and large number of preprint articles, our findings from the first eight of the pandemic in over 15,000 COVID-19 cases suggested an incremental efficacy of tocilizumab in severe COVID-19 that were confirmed by subsequent meta-analyses of large randomized trials of tocilizumab. This suggests that analysis of case-control studies and pre-print server data in the early stages of a pandemic appeared robust for supporting incremental benefits and lack of major therapeutic toxicity of tocilizumab for severe COVID-19.

## 1. Introduction

“Severe Acute Respiratory Syndrome Coronavirus type 2” (SARS-CoV-2) has been identified as the infectious agent responsible for the potentially life-threatening “Coronavirus disease 2019” (COVID-19) [1]. Since December 2019, this virus has quickly spread out from China, becoming a global pandemic, with high death rates [1,2,3].

In the absence of proven antiviral or vaccine strategies, with vaccines being approved only recently, there has been considerable interest in the dysregulated immune response accompanying SARS-CoV-2, since poor prognosis has been repeatedly shown to correlate with elevation of inflammatory markers [4,5,6]. Indeed, the analogy with a hypercytokinaemic state or cytokine storm that typically occurs in macrophage activation syndrome (MAS) has been made [7]. In the cytokine storm or MAS states, including those linked to chimeric antigen receptor T-cell therapy (CAR-T) or Still’s disease, impressive responses have been reported with anti-cytokine therapy against interleukin 6 (IL-6) or interleukin 1 (IL-1) [8,9].

Tocilizumab is a monoclonal antibody targeting both the soluble and membrane-bound forms of the IL-6 receptor (IL-6R) [10]. By blocking IL-6R, tocilizumab prevents the *cis*- and *trans*-activation of the JAK-STAT pathway as well as the MAPK/NFκB cascade and other networks triggered by IL-6, with consequent broad antagonism of both innate and adaptive immunity [10]. Tocilizumab is licensed for the treatment of rheumatoid arthritis (RA), systemic-onset polyarticular juvenile idiopathic arthritis (JIA), giant cell arteritis (GCA), and severe/life-threatening cytokine storm also known as cytokine release syndrome (CRS) secondary to the use of CAR-T cell therapy [11,12]. Of note, subjects with severe COVID-19 pneumonia have been reported to have higher levels of detectable serum IL-6 levels [13].

In the absence of definitive COVID-19 therapies in the face of a severe cytokine storm, tocilizumab has been proposed as a potential treatment, especially in individuals exhibiting high levels of inflammatory markers. Consequently, tocilizumab therapy has been at the vanguard of biological therapy for severe COVID-19 pneumonia with highly impressive initial reports from open-label studies from China [14]. However, some investigators have sounded a word of caution that the immune activation in COVID-19 is a lung specific immunopathology that may be reactive to an active ongoing viral pneumonitis and, unlike the CAR-T/Still’s disease setting, the impact of tocilizumab, even though promising [15], is far from clear. In addition, in Rheumatology practice, tocilizumab therapy is typically reserved for subjects that fail to adequately respond to disease-modifying antirheumatic drug (DMARDs) or require ongoing high dose chronic corticosteroid administration. However, chronic corticosteroid use is not needed for COVID-19 pneumonia and, in the face of an escalating pandemic and encouraging results from corticosteroid therapy, there is an urgent need to define the optimal use, if any, of tocilizumab in severe COVID-19 MAS pneumonia [16,17].

Therefore, the purpose of the present systematic review and meta-analysis was to investigate whether tocilizumab therapy directed against severe COVID-19 improved survival. We investigated its effectiveness both in intensive care unit (ICU) and non-ICU settings, both in ventilated and not ventilated patients, and evaluated the impact of concomitant corticosteroid therapy on survival. With the sheer volume of rapidly appearing publications in this arena, we also stratified our analysis for an independent evaluation of peer reviewed and pre-print publications.

As tocilizumab use is associated with an increased risk of bacterial infections in the Rheumatology arena, we also evaluated its safety profile.

## 2. Material and Methods

### 2.1. Systematic Review Study Protocol and Systematic Review Findings Reporting

The study protocol was devised according to the “Preferred Reporting Items for Systematic reviews and Meta-Analyses–Protocol” (PRISMA-P) guidelines [18]. The findings are here reported according to the “Preferred Reporting Items for Systematic reviews and Meta-Analyses” (PRISMA) guidelines [19]. The systematic review and meta-analysis study protocol has been registered with PROSPERO Number CRD42020195690.

### 2.2. Search Strategy

The following string of keywords was searched: “tocilizumab AND (2019-nCoV OR COVID-19 OR SARS-CoV-2)”. Synonyms were used as well, such as “ACTEMRA”, “IL-6 blocker” or “IL-6 blockade therapy” for tocilizumab and “novel coronavirus”, “emerging coronavirus” or “Wuhan coronavirus”, for the infectious agent. No time or language filters were applied. PubMed/MEDLINE and Scopus were extensively mined from inception until 20 July 2020, together with pre-print servers, namely medRxiv, Research Square and SSRN.

### 2.3. Inclusion and Exclusion Criteria

The following PICOS criteria were considered:

- P—(patients): subjects with COVID-19 (either suspected or confirmed);
- I—(intervention): treated with tocilizumab;
- C—(comparator/comparison/control): any kind of comparison possible (such as tocilizumab versus tocilizumab plus standard care, one versus multiple doses, intravenous versus subcutaneous injection, earlier versus later administration, administration in hospital ward versus in ICU setting, in ventilated versus not ventilated patients, and use of other concomitant therapy);
- O—(outcomes): mortality rate, admission to the ICU, need for mechanical ventilation, impact of concomitant therapy use on survival, with an emphasis on corticosteroids, and side-effects;
- S—(study design): investigations designed as case-control studies, ether matched or unmatched, and those investigations that, even if not explicitly devised as case-control studies, provided information for each treatment cohort.

Original investigations designed as case reports, case series and cross-sectional studies not providing information for each treatment cohort, as well as studies devised as editorials, letters to editor, commentaries, and reviews (of any type) were excluded. 

### 2.4. Data Extraction

Relevant data were independently extracted by two researchers (A.W. and N.L.B.): namely, reference, country in which the study was conducted, sample size (overall number of patients, those receiving tocilizumab plus standard care and those receiving standard care), inclusion and exclusion criteria, main demographic characteristics of the recruited sample, including age, sex, underlying co-morbidities, treatment received (tocilizumab dosage/schedule, starting of tocilizumab in relationship to time of admission, concomitant therapies such as antibiotics, antiviral medications including ritonavir/lopinavir or remdesivir, steroids and anticoagulants). 

The following parameter(s)/outcome(s) were evaluated; clinical and laboratory parameters, admission to the ICU, need for mechanical ventilation, and side effects. Disagreements were resolved by discussion with the senior author (D.M.G.) until consensus was achieved. 

Considering the emergency nature of the situation and given that tocilizumab has been administered in a compassionate, off-label way, and was not available in every healthcare facility, due to the general shortage of drugs, it was not always possible to perform a rigorous patient enrolment by applying stringent screening and inclusion/exclusion criteria. As such, most studies presented statistically significant differences between cases and controls, which calls up for caution when interpreting univariate, unadjusted results. To cope with these issues, we assessed whether the study authors adjusted for the outcomes, for example performing propensity-based inverse probability weighting models or multivariate analyses. Further details concerning research strategy are reported in Table 1.

### 2.5. Study Quality Appraisal

Quality assessment was performed independently by two researchers (A.W. and N.L.B.) by means of the Newcastle–Ottawa scale for case-control studies. This instrument comprises eight items investigating domains such as selection (four stars), comparability (two stars) and exposure (four stars). Specifically concerning comparability, one star was awarded in case of matching for socio-demographic parameters and a further star was given in case of homogenous pharmacological treatment between cases and controls. 

### 2.6. Statistical Analysis

Prevalence rates, odds-ratios (ORs) and hazard-ratios (HRs) were pooled together utilizing classical meta-analytical approaches. In more detail, when authors did not provide an already computed effect size, numbers of events for each outcome were extracted both for cases (patients receiving tocilizumab plus standard care) and controls (receiving standard care only) to compute the combined effect size together with its 95% confidence interval (CI). When authors provided an already computed effect size (generally adjusted), these were combined together. Based on the Q and I^2^ tests, depending on the amount of heterogeneity among studies, a random-effects model was preferred over a fixed-effects one [20,21]. For each outcome of interest, we present the overall combined effect size based on the 2 × 2 contingency table (unadjusted effect size), the combined effect size pooling together only those studies which performed case-control matching (adjusted effect size) and the various results at the single study level. In case of a significantly high amount of heterogeneity among studies, meta-regressions and sub-group analyses were performed to shed light on the determinants of such heterogeneity.

The presence of publication bias was assessed by visually inspecting the funnel plot and carrying out the Egger’s linear regression test. All analyses were conducted with the commercial software “Comprehensive Meta-Analysis” (CMA version 3.0, for Windows, Biostat, Englewood, NJ, USA).

## 3. Results

### 3.1. Search Strategy and Study Setting

**Figure 1:**
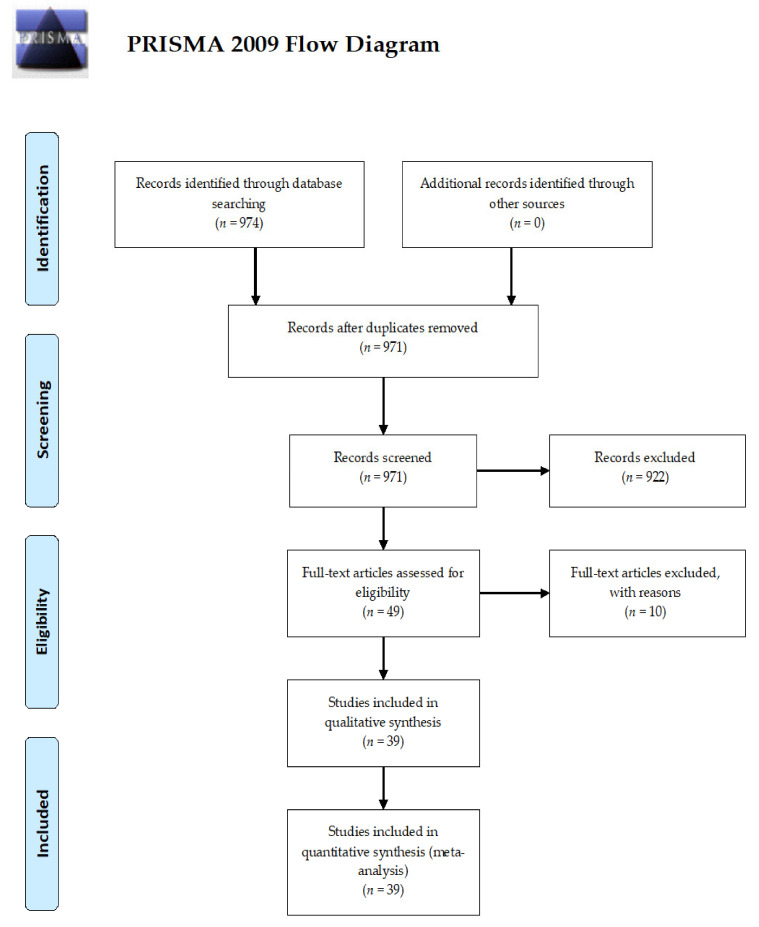
The study retrieval and selection process adopted in the present systematic review and meta-analysis.

The initial search yielded a pool of 974 items with 39 studies included in the present systematic review and meta-analysis, totalling a sample of 15,531 patients (3657 cases versus 11,874 controls) [22,23,24,25,26,27,28,29,30,31,32,33,34,35,36,37,38,39,40,41,42,43,44,45,46,47,48,49,50,51,52,53,54,55,56,57,58,59,60]. The process of retrieval, inclusion and exclusion process is pictorially shown in Figure 1. The main characteristics of the studies retained are presented in Table 2 and Table 3. Of the 39 studies included, one [28] was exclusively devoted to the safety profile of tocilizumab, only 15 (38.5%) carried out propensity score-based inverse probability treatment weighting models or performed adjustments for mismatching between cases and controls by means of multivariate analyses. At the time of the search, 67% (*n* = 26) of the investigations retained were released as pre-prints.

### 3.2. Age and Gender in Tocilizumab Treated Cases and Controls

For 14 studies, no information was available regarding age. Mean age among cases ranged from 55 to 76.8 years. Of the included studies, slightly more than half (52.0%) were age mismatched between cases and controls, especially those with higher sample-sizes, a key consideration given that older age is linked to COVID-19 mortality. In 11 studies the cases and controls were aged matched and in three studies tocilizumab treated cases were older than controls (even though in one of these studies the difference did not achieve statistical significance), while in eleven studies the controls were older than tocilizumab treated cases. To see if age mismatching impacts on the outcomes of interest, meta-regressions and sub-group analyses were performed. Concerning gender distribution, overall, this did not differ between cases receiving tocilizumab and controls receiving standard care. In 10 studies, among cases there were less females than among controls. No other significant differences concerning other socio-demographic parameters could be found. A more detailed description of this data is found in Table 2 and Table 3.

### 3.3. Underlying Co-Morbidities

Overall, the rate of underlying co-morbidities was comparable between those treated with tocilizumab and controls in this 15,000-patient group study with only eight studies exhibiting differences between cases and controls (See Table 2 and Table 3). These studies were generally small-medium size, with only one large-size.

### 3.4. Laboratory Parameters and Oxygen Saturation

Markers including CRP, D-dimer, CK, troponin-T and LDH level elevations are associated with poor prognosis in COVID-19 pneumonia and these collectively are thought to represent virally mediated immune-mediated thrombosis with hypoxemia with associated cardiac stress and increased mortality [7]. In the 39 studies, when reported, tocilizumab cases generally displayed worse prognostic biomarkers, such as higher IL-6 and CRP levels compared to controls. The overall oxygen saturation and PaO_2_/FiO_2_ ratio were lower in the tocilizumab cases group pre-therapy than in controls. Collectively these poor prognostic features were over-represented in the tocilizumab treated groups (Table 2 and Table 3).

### 3.5. Concomitant Therapies

Three studies did not provide information about standard care therapy; hydroxychloroquine was administered in 36 studies, antivirals and antiviral agents were provided in 27 studies and corticosteroids use was reported in 30 studies, without necessarily referring to posology or route of administration, thus complicating a detailed analysis of the impact of corticosteroids. Antibiotics were delivered in 36 studies, whereas anti-coagulants were administered in 12 studies. Generally, there was no difference in terms of drug use between the two groups. The three studies where tocilizumab was administered without steroids were otherwise balanced for the aforementioned alternative therapies. 

### 3.6. Major Outcomes

#### 3.6.1. Overall Impact of Tocilizumab on Death Rate

**Figure 2:**
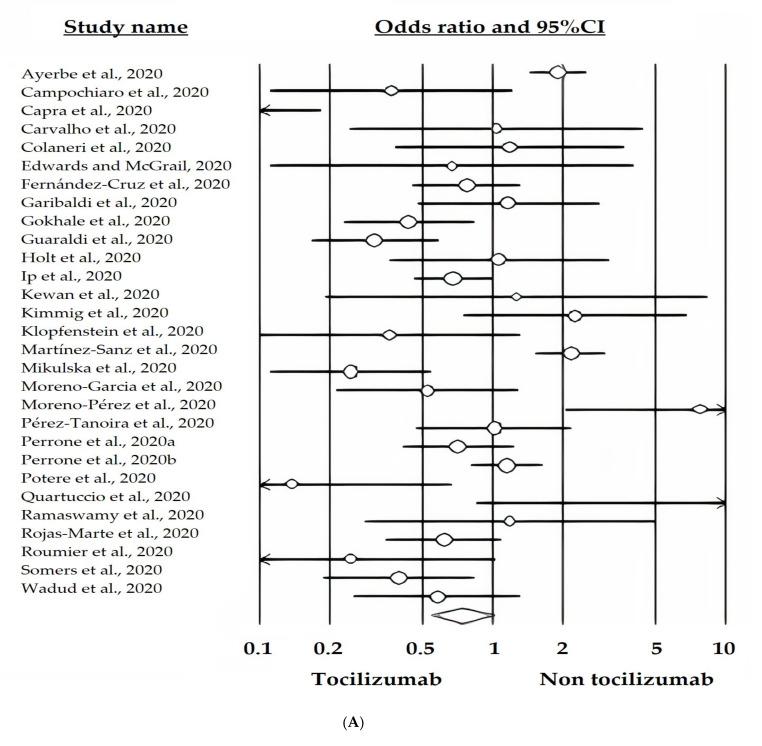

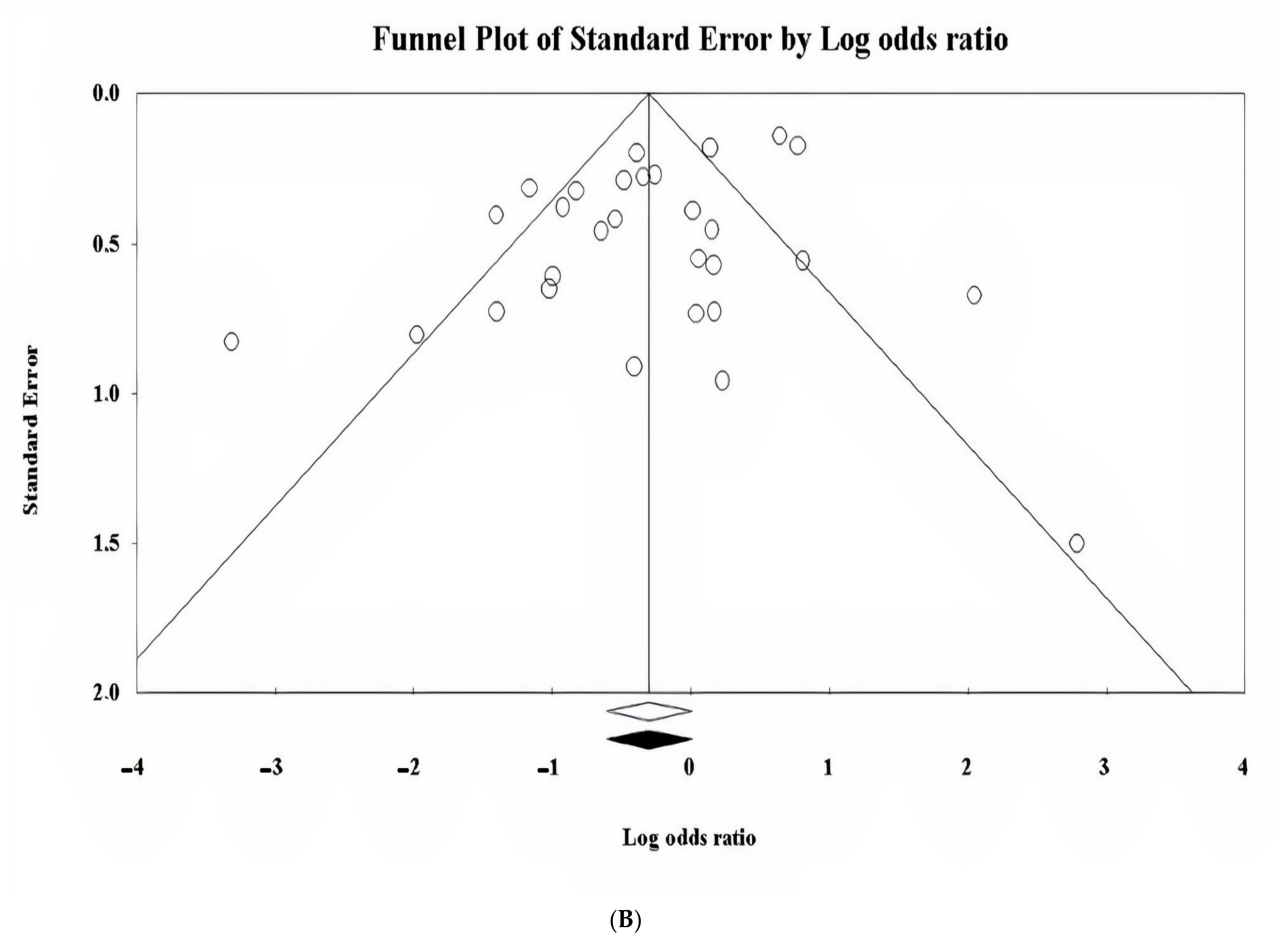

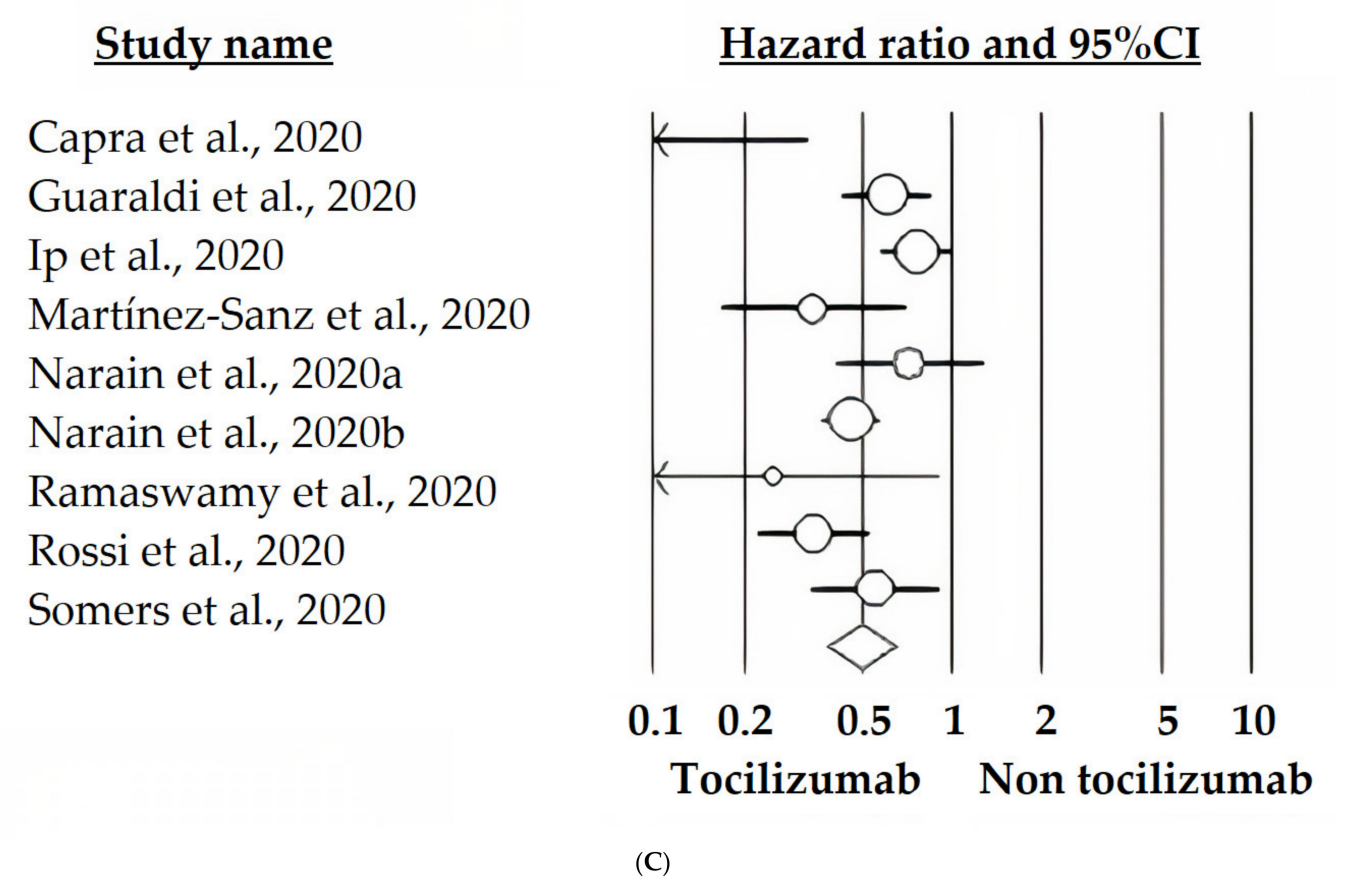

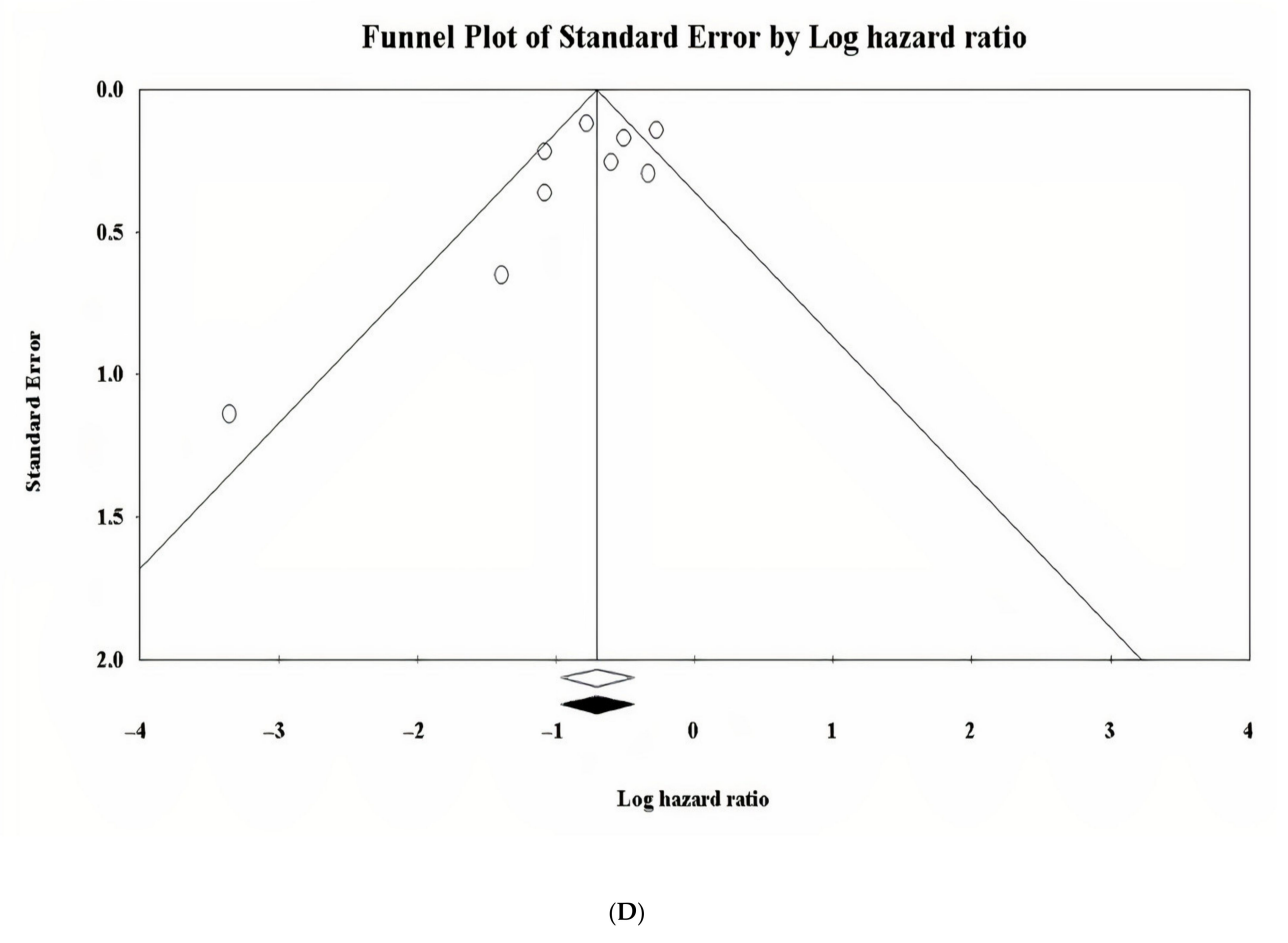

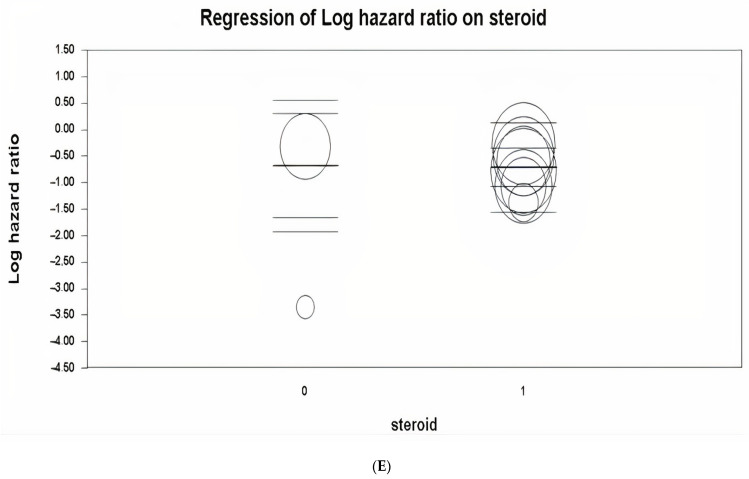
(**A**). Random-effects model forest plot of the impact of Tocilizumab on death rate in cases receiving tocilizumab plus standard care versus controls receiving standard care, according to studies reporting unadjusted estimates (crude events). The forest plot shows a high amount of heterogeneity among studies and a borderline protective effect of tocilizumab. (**B**) Funnel plot of the random-effects model of the impact of Tocilizumab on death rate in cases receiving tocilizumab plus standard care versus controls receiving standard care, according to studies reporting unadjusted estimates (crude events). It shows no evidence of publication bias. (**C**) Random-effects model forest plot of the impact of Tocilizumab on death rate in cases receiving tocilizumab plus standard care versus controls receiving standard care, according to studies reporting adjusted estimates. The forest plot shows a high amount of heterogeneity among studies and a protective effect of tocilizumab. (**D**) Funnel plot of the random-effects model of the impact of Tocilizumab on death rate in cases receiving tocilizumab plus standard care versus controls receiving standard care, according to studies reporting adjusted estimates. It shows no evidence of publication bias. (**E**) Meta-regression showing no statistically significant difference between the use of tocilizumab plus standard care i.e., without steroid (0) and the concomitant use of steroids (1) in terms of mortality rate.

Unadjusted estimates (*n* = 28, k = 29) failed to demonstrate a protective effect of tocilizumab: OR 0.74 ([95%CI 0.55–1.01], *p* = 0.057; Q = 136.58, DF = 28, I^2^ = 79.50) (Figure 2A). At the meta-regressions level, no effects of study quality (selection *p* = 0.2629, comparability *p* = 0.2227, and exposure *p* = 0.2684) or publication status (pre-prints versus published in peer-reviewed journals; *p* = 0.4376) could be detected. No evidence of publication bias could be found (Figure 2B).

The number of studies was substantially reduced on pooling together the studies reporting adjusted effect sizes (*n* = 8 studies, k = 9), applying the random-effects model due to the significant amount of heterogeneity among studies (Q = 21.98, DF = 8, I^2^ = 63. 61%), tocilizumab therapy showed an overall protective effect with an HR of 0.50 ([95%CI 0.38–0.64], *p* < 0.001) (Figure 2C). No effect of publication status (pre-print versus published in peer-reviewed journal; *p* = 0.9346) could be detected, as well as no publication bias (Figure 2D). 

#### 3.6.2. Impact of Concomitant Corticosteroid Use on Survival

To study the impact of concomitant use of steroids, an exploratory meta-regression was conducted. Results of the meta-regression (*p* = 0.9450) indicated that tocilizumab administration was effective in both treatment cohorts, that is to say in those utilizing steroids and in those to whom corticosteroids were not administered. Tocilizumab with concomitant steroid use versus standard care, that may have also included steroid was protective with an OR 0.49 ([95%CI 0.36–0.65], *p* < 0.05), but it was not possible to accurately evaluate steroid use between the two groups. Tocilizumab alone (without steroid) versus standard care without steroid also appeared protective OR 0.59 ([95%CI 0.34–1.00], *p* < 0.001) in three studies (Figure 2E). 

#### 3.6.3. Impact of Tocilizumab on Preventing Mechanical Ventilation

**Figure 3:**
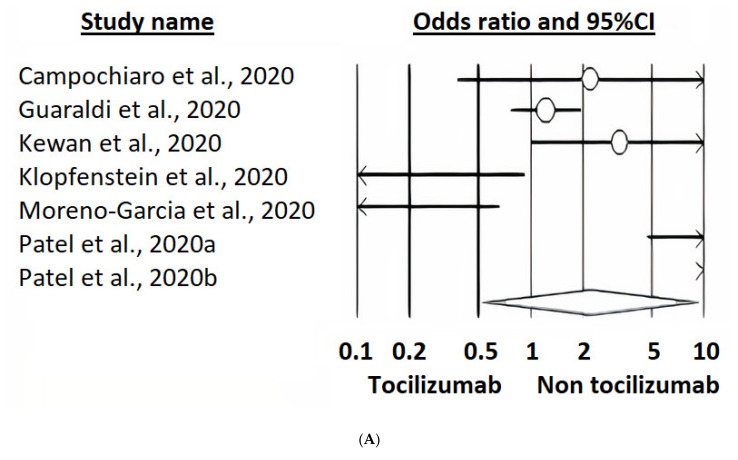

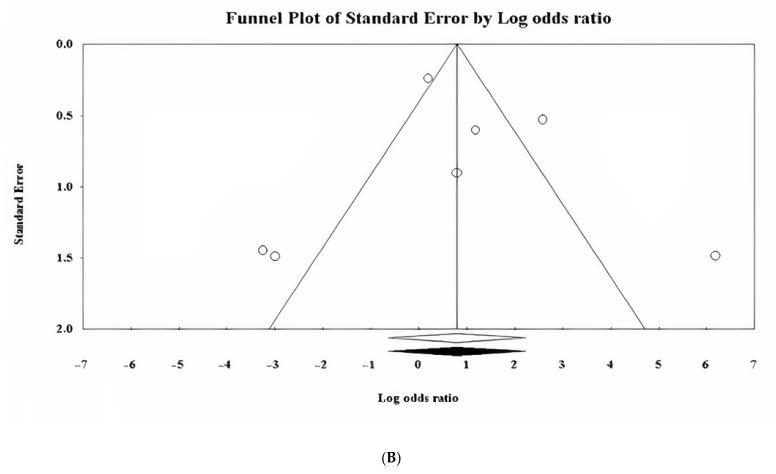
(**A**) Random-effects model forest plot of the impact of tocilizumab on the need for mechanical ventilation in cases receiving tocilizumab plus standard care versus controls not receiving tocilizumab, according to studies reporting unadjusted estimates (crude events). It shows a high amount of heterogeneity among studies and no effect of tocilizumab on ICU admission rate. (**B**) Funnel plot of the random-effects model of the impact of tocilizumab on the need for mechanical ventilation in cases receiving tocilizumab plus standard care versus controls not receiving tocilizumab, according to studies reporting unadjusted estimates. It shows no evidence of publication bias.

Unadjusted estimates (*n* = 6, k = 7) showed no protective effect of tocilizumab on preventing mechanical ventilation (OR 2.21 [95%CI 0.53–9.23], *p* = 0.277; Q = 44.67, DF = 6, I^2^ = 86.57) (Figure 3A). At the meta-regressions, only selection impacted size (*p* = 0.0008), whereas comparability and exposure had no effects (*p* = 0.1565 and *p* = 0.1197, respectively). No evidence of publication bias could be found (Figure 3B) and neither could any effect of publication status (pre-print versus published in a peer-reviewed journal, *p* = 0.2034). However, the only study reporting adjusted estimates (the investigation by Rossi et al.) displayed a protective effect (HR 0.49 [95%CI 0.30–0.81], *p* < 0.01).

#### 3.6.4. Studies Specifically Reporting the Impact of Tocilizumab on ICU Admission

**Figure 4:**
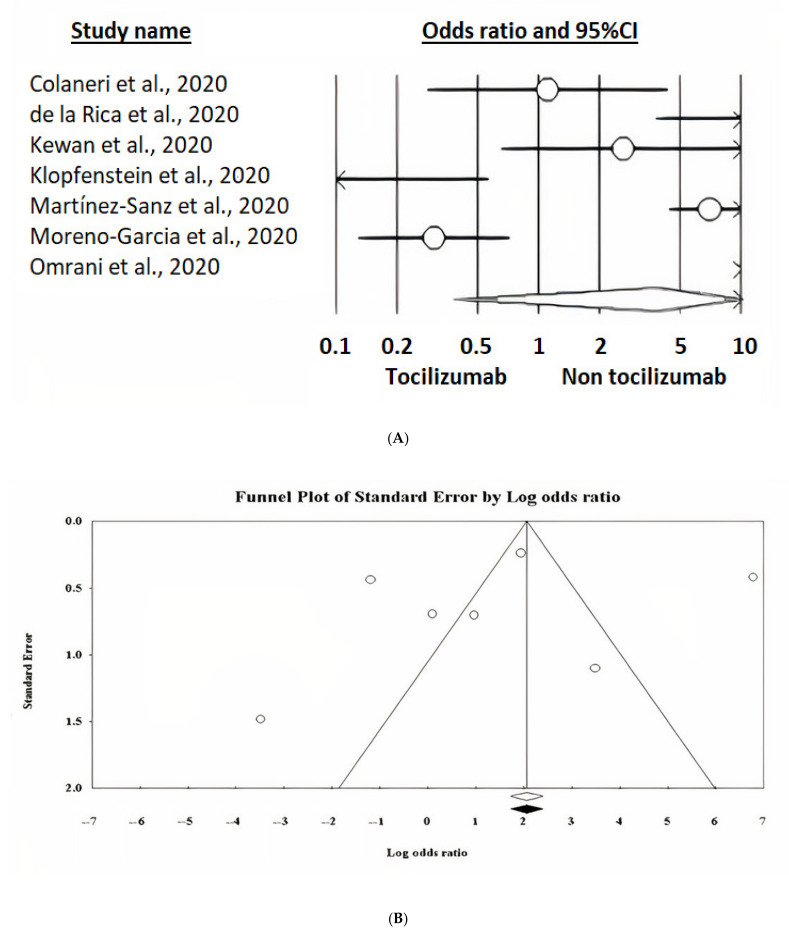

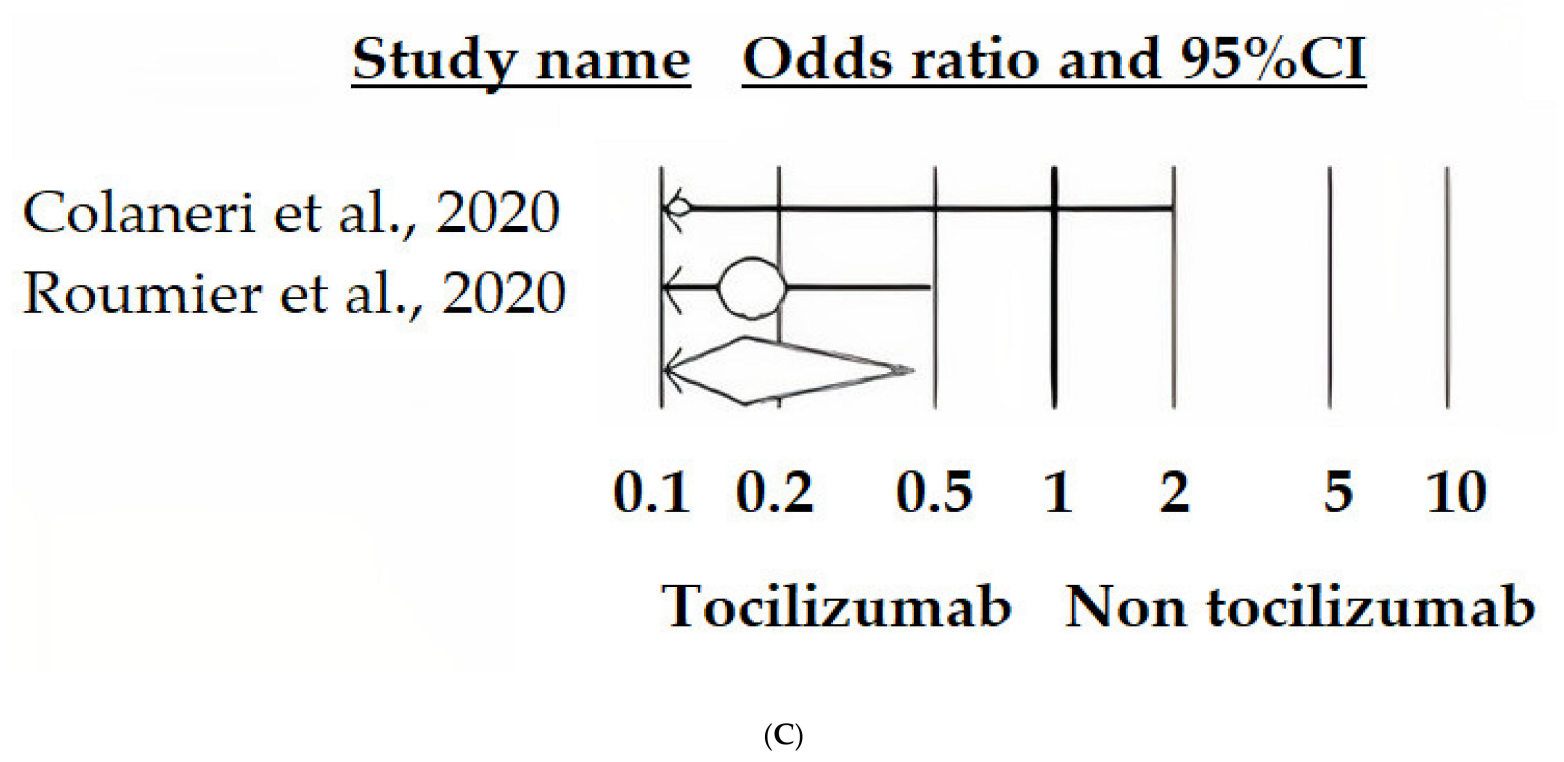
(**A**) Random-effects model forest plot of the impact of tocilizumab on ICU admission rate in cases receiving tocilizumab plus standard care versus controls not receiving tocilizumab, according to studies reporting unadjusted estimates (crude events). It shows a high amount of heterogeneity among studies and no effect of tocilizumab on ICU admission rate. (**B**) Funnel plot of the random-effects model of the impact of tocilizumab on ICU admission rate in cases receiving tocilizumab plus standard care versus controls not receiving tocilizumab, according to studies reporting unadjusted estimates. It shows no evidence of publication bias. (**C**) Fixed-effects model forest plot of the impact of tocilizumab on ICU admission rate in cases receiving tocilizumab plus standard care versus controls not receiving tocilizumab, according to studies reporting adjusted estimates. It shows no heterogeneity among studies and a protective effect of tocilizumab on ICU admission rate.

Unadjusted estimates showed no protective effect (OR 3.79 [95%CI 0.38–37.34], *p* = 0.254) (Figure 4A). No effect of publication bias could be found (Figure 4B) as well as no effect of publication status (pre-print versus published in a peer-reviewed journal, *p* = 0.1627). However, two studies reported adjusted estimates of the impact of tocilizumab on reduction of ICU admission. Applying a fixed-effects model, pooling these two studies the combined effect size resulted in OR 0.16 ([95%CI 0.06–0.43], *p* < 0.001) (Figure 4C).

#### 3.6.5. Tocilizumab Side-Effects

Unadjusted estimates showed an increased risk of bacterial infections, with an OR of 2.36 [95%CI 1.001–5.54], *p* = 0.050 (Q = 74.73, DF = 9, I^2^ = 87.96). Only the study by Carvalho et al. [26] reported adjusted estimates, with an OR 1.73 ([95%CI 0.22–13.82], *p* = 0.6). 

## 4. Discussion

A subgroup of COVID-19 patients develops severe pneumonia with some features of a cytokine storm, which may contribute to patient mortality [61,62]. The emergent evidence of corticosteroid efficacy in severe COVID-19 disease in both open and controlled trials strongly attests to a pulmonary macrophage activation syndrome-like disease dramatically impacting on mortality [16,17]. Interleukin-6 and several other cytokines are pivotal to the immunopathogenesis of cytokine storm, and IL-6 elevations have been reported in some severe COVID-19 studies [7,63]. The COVID-19 pandemic is still escalating and treating physicians desperately need knowledge on the optimal use of corticosteroids and cytokine blockers, or both in combination for severe COVID-19 disease. This meta-analysis in over 15,000 COVID-19 using unadjusted estimates failed to show a reduction in mortality with tocilizumab, although a trend for reduced mortality was evident. An unadjusted analysis also failed to show a protective effect of tocilizumab on preventing mechanical ventilation and also ICU admission. According to adjusted models where patient numbers were much smaller, tocilizumab use was associated with an overall mortality reduction.

The data pertaining to the efficacy of tocilizumab or otherwise as an adjunct therapy added to corticosteroids were unclear at the start of the pandemic. In this metanalysis, accurate description of steroid dose between the tocilizumab and standard of care groups was not available which made data difficult to interpret. 

During the first eight months of the pandemic, we became aware of a negative phase three randomised controlled trial of tocilizumab where we understand that corticosteroid was also part of the control group. The issue of the impact of corticosteroids needed further evaluation in the trial arena but had large health economic cost implications if the addition of tocilizumab to corticosteroid therapy improved survival. Indeed, we found some small trials that supported the efficacy of tocilizumab monotherapy where steroids were not part of the standard of care group [24,25,57].

Despite the heterogeneity of included studies and large number of preprint articles, our findings from the first eight of the pandemic in over 15,000 COVID-19 cases suggested an incremental efficacy of tocilizumab in severe COVID-19 that were confirmed by subsequent meta-analyses of large randomized trials of tocilizumab [64,65,66]. This suggests that analysis of case-control studies and pre-print server data in the early stages of a pandemic appeared robust for supporting incremental benefits of tocilizumab for severe COVID-19.

In this meta-analysis, tocilizumab use was associated with an increased the risk of bacterial infection, but we could not link this to an increased mortality. Unlike the emergent data from the RECOVERY trial [16] where the beneficial impact of corticosteroid therapy was highest in ventilated cases, the available data in this meta-analysis precluded a specific analysis of the impact of tocilizumab therapy on mortality by patient stratification according to mechanical ventilation in ICU settings versus non-ICU.

Since the start of the COVID-19 pandemic, it has emerged that the immunopathogenesis of the accompanying cytokine storm may at least in part be due to unrestrained SARS-CoV2 replication due to blunting of both innate type-1 interferon responses and adaptive immune responses with severe lymphopenia and T-cell functional exhaustion. Indeed, use of high dose steroids in non-severe COVID-19 disease could potentially be detrimental to survival [16]. Consequently, IL-6R blocking might in some circumstances be counterproductive and could theoretically exacerbate severe disease. 

Reassuringly, there was no evidence from our systematic review and meta-analysis of an increased mortality related to tocilizumab therapy, but optimal therapy especially in subjects with ongoing viral replication still needs to be defined. Furthermore, many ICU ventilated cases have acute respiratory distress syndrome (ARDS) which in severe cases may be linked to IL-6 elevations and given the role of IL-6 in tissue repair, the outcome of tocilizumab on ventilated ARDS cases is worthy of consideration. The toxicities of very high doses of steroids, including the induction of avascular necrosis in patients with severe hypoxaemia, and other steroid toxicities are avoided, which adds further reassuring data to the safety of cytokine antagonism.

Despite the inconclusiveness of some of the present data and the consistently high heterogeneity among studies included, information provided could be meaningful both for identifying an optimal patient/candidate for tocilizumab based treatment and for devising future treatment. Tissue repair will be a major issue in COVID-19 pneumonia. IL-6 can act on tissue remodelling and injury, and the timing and amount of antibodies to suppress injury can be an issue. In addition, IL-6 suppression does not resolve all of the aspects of the multifaceted pathophysiology of COVID-19 pneumonia, including cell death, abnormal coagulation and lung inflammation caused by viral infection.

The present systematic review and meta-analysis is not without shortcomings. The heterogeneity was high for all outcomes of interest, which represents a limitation of our study. However, this issue was addressed by means of meta-regressions and subgroup analyses to shed light on the determinants of such heterogeneity. Another major limitation is given by the inclusion of studies which, lacking of proper controls, do not perform adjustments for confounding factors, therefore masking the real potential effect of tocilizumab administration. Generally, the tocilizumab therapy group was younger than the control group, but this was balanced by a greater magnitude elevation in inflammatory makers and underlying co-morbidities. However, the effect of each co-variate, including age, gender, and co-morbidities, was assessed by means of meta-regressions and subgroup analyses. Furthermore, estimates derived from pooling together only those studies which carried out corrections for mismatching, despite representing a subset of all investigations retained totalling a lower sample size of patients, are more reliable and statistically robust, which represents a strength of our study, and has been confirmed by subsequent RCTs. Finally, we also included several studies awaiting for formal peer review, which could also represent a study limitation, even though meta-regression analysis showed no statistically significant differences between peer-reviewed articles and pre-prints. Hence, the peer reviewed published articles show a protective effect of tocilizumab in COVID-19 pneumonia.

It is important to mention that controls were treated with standard-of-care treatments, including hydroxychloroquine, azithromycin, antiviral therapy and heparin in most cases. In this meta-analysis, all studies (with the exception of three studies) included antivirals and corticosteroids in both the tocilizumab and standard-of-care group. Recent press release and preprint server publications showed a greater survival for corticosteroids over standard of care in ventilated cases over non-ventilated ill COVID-19 patients, but we were unable to accurately interrogate the data to evaluate this issue with respect to tocilizumab administration since it was often unclear in what setting tocilizumab was administered. Furthermore, the impact on antiviral therapy in limiting SARS-CoV2 replication in the face of steroid and tocilizumab therapy needs consideration as a potential factor that may contribute to the apparent benefit of tocilizumab. Although in some studies, certain disorders such as hypertension and chronic pulmonary disease were more prevalent among those treated with tocilizumab than controls, the overall rate of underlying co-morbidities was comparable between the two groups.

In conclusion, this systematic review and meta-analysis of case-control studies and unadjusted analysis in large numbers of cases failed to show a benefit of tocilizumab in a real-world setting type scenario. However, adjusted analysis in smaller numbers showed that tocilizumab may reduce death rates in severe COVID-19.

These preliminary findings including case control and pre-print server studies were subsequently confirmed by large RCTs and meta-analysis of these RCTs that showed incremental benefit with tocilizumab [64,65,66]. Therefore, our systematic literature review and meta-analysis strategy at the start of the pandemic with large numbers of cases point towards the robustness of such a strategy early in the face of the pandemic. Summarizing, early use of case control studies including non-peer reviewed data seem to provide valuable information in the face of a rapidly escalating pandemic.

## Figures and Tables

**Table 1:** Details of the search strategy adopted in the present systematic review and meta-analysis.

Systematic Review Search Strategy Item	Details
International scholarly electronic databases searched	PubMed/MEDLINE, Scopus, pre-print servers (medRxiv, SSRN, Research Square)
Keywords	(“SARS-CoV-2” OR “novel coronavirus” OR “emerging coronavirus” OR “Wuhan coronavirus” OR “2019-nCoV” OR “COVID-19”) AND (tocilizumab OR Actemra OR “interleukin 6-blockade treatment” OR “interleukin 6-blockade therapy” OR “IL-6-blockade treatment” OR “IL-6-blockade therapy” OR “IL-6 blocker”)
Time filter	None
Language filter	None
Inclusion criteria	P (patients): laboratory- and/or radiologically suspected or confirmed COVID-19 patients I (intervention): treated with tocilizumab C (comparisons/comparators/controls): pharmacological treatment (tocilizumab + standard care versus standard care); dose, route, timing, and setting of tocilizumab administration, and use of concomitant therapy O (outcomes): death rate, need for mechanical ventilation; ICU admission, side-effects S (study design): any original paper (designed as case report)
Exclusion criteria	P (patients): patients without a suspected/confirmed diagnosis of COVID-19 I (intervention): other treatments rather than tocilizumab C (comparisons/comparators/controls): comparisons different from those previously stated (for example, disease severity) O (outcomes): outcomes different from those previously stated or reporting those outcomes without sufficient details S (study design): any kind of review paper (including systematic reviews and meta-analyses if available), letter to editor, editorial, commentary, expert opinion; original studies designed as case reports, case series, cohort studies
Hand-searched target journals	Any journal potentially related to intensive care medicine, infectious disorders, virology, microbiology, epidemiology, global and public health, hygiene

**Table 2:** Main characteristics of included studies.

Reference	Country	Sample Size	Inclusion Criteria	Age	Sex	Treatment	Tocilizumab Dosage	Starting of Tocilizumab	Admission to ICU	Parameters/Outcomes Evaluated
Allenbach et al., 2020 [22]	France	147 consecutive patients out of an initial list of 152 (6 cases and 141 controls)	Laboratory-confirmed cases (positive SARS-CoV-2 RT-PCR assay from nasal swabs)	NA	NA	Tocilizumab + standard care (hydroxychloroquine, antibiotics and steroids)	NA	At admission	Hospitalized; with some admitted to the ICU	Composite index (mortality rate and/or ICU admission)
Ayerbe et al., 2020 [23]	Spain	2019 consecutive patients (421 cases and 1598 controls); all severe	Laboratory-confirmed cases	66.1 ± 13.11; younger than controls (*p* = 0.0267)	*n* = 304 (72.21%); less females among cases than among controls	Tocilizumab + standard care (hydroxychloroquine, azithromycin, steroids, lopinavir/ritonavir, or oseltamivir, heparin)	NA	NA (at any time during admission)	All hospitalized; none admitted to the ICU	Mortality rate (after 8 days of follow–up)
Campochiaro et al., 2020 [24]	Italy	65 consecutive patients (32 cases versus 33 controls), with severe disease	Laboratory-and radiologically-confirmed cases	64 [range 53–75]; no difference with controls	*n* = 29 (91%); no difference with controls	Tocilizumab + standard care (hydroxychloroquine, lopinavir/ritonavir, ceftriaxone, azithromycin, anti-coagulation prophylaxis with enoxaparin)	Single dose of i.v. 400 mg followed by a dose of 400 mg 24 h after in case of respiratory worsening. A second dose was administered in 9 (28%) patients (seven of which were under non-invasive ventilation)	24 h prior to ICU admission and/or intubation	Hospitalized and all admitted to the ICU; 25 (78%) under non-invasive ventilation, 4 (13%) under mechanical ventilation	Mortality rate (at 28 days) Need for ventilation
Capra et al., 2020 [25]	Italy	85 consecutive patients (62 cases versus 23 controls)	Laboratory- and radiologically-confirmed cases; critically ill patients requiring mechanic ventilation, with abnormal platelets and transaminases values were exclusion criteria	63 [range 54–73]; younger than controls	45(73%); less males among cases than among controls (73% versus 83%)	Tocilizumab + standard care versus standard care (hydroxychloroquine and lopinavir plus ritonavir)	33 (53%) 400 mg i.v. once, 27 (43.5%) subcutaneous 324 mg once; 2 (3.5%) 800 mg i.v.	As soon as tocilizumab was available (within 4 days from admission)	Hospitalized, no one admitted to ICU, 5 under mechanical ventilation	Mortality
Carvalho et al., 2020 [26]	Brazil	53 consecutive patients (29 cases and 24 controls); all critically ill	Suspected or laboratory-confirmed cases	55 [range 44–65]	62%	Tocilizumab + standard care (hydroxychloroquine, azythromycin, steroids)	400 mg i.v., two doses	At admission	Admitted to the ICU	Mortality Positive cultures
Colaneri et al., 2020 [27]	Italy	112 patients from the SMACORE study (21 cases and 91 controls)	Laboratory-confirmed cases	62.33	19/21 (90.5%); less females among cases than among controls	Tocilizumab + standard care versus standard care (hydroxychloroquine, azithromycin and low weight heparin, and methylprednisolone)	8 mg/kg (up to a maximum 800 mg per dose) i.v., repeated after 12 h	NA	Hospitalized, 3 admitted to the ICU	ICU admission Mortality
Crotty et al., 2020 [28]	USA	289 patients (18 cases and 271 controls)	Laboratory-confirmed cases	NA	NA	Tocilizumab + standard care (antibiotics, hydroxychloroquine, remdesivir, steroids)	NA	NA	Hospitalized patients	Infections
de la Rica et al., 2020 [29]	Spain	58 patients (11 and 47 controls)	Laboratory-confirmed cases (nasal and pharyngeal swabs)	NA	NA	Tocilizumab + standard care (chloroquine or hydroxychloroquine, Remdesivir, lopinavir + ritovanir, steroids, antibiotics, interferon beta)	NA	NA	Hospitalized patients	ICU admission
Edwards and McGrail, 2020 [30]	USA	35 consecutive patients (11 cases and 24 controls), all critically ill	Laboratory-confirmed cases	NA	NA	Tocilizumab + standard care (hydroxychloroquine, azithromycin and convalescent plasma; remdesivir only for 1 patient)	4/11 receiving a second dose	NA	Admitted to the ICU; 8/11 requiring mechanical ventilation	Mortality
Fernández-Cruz et al., 2020 [31]	Spain	463 (180 cases and 283 controls)	Laboratory-confirmed cases	NA	NA	Tocilizumab + standard care (hydroxychloroquine, lopinavir/ritonavir, antibiotics, interferon)	NA	NA	NA	Mortality rate
Garibaldi et al., 2020 [32]	USA	832 patients (39 cases and 793 controls)	Laboratory-confirmed cases	NA	NA	Tocilizumab + standard care (antibiotics, hydroxychloroquine, corticosteroids, antivirals)	NA	NA	NA	Mortality rate
Gokhale et al., 2020 [33]	India	161 consecutive patients (70 cases and 91 controls)	Laboratory-confirmed cases	52 [range 44–57], younger than controls (*p* = 0.001)	67.1%	Tocilizumab + standard care (antibiotics, hydroxychloroquine, ivermectin, oseltamivir, low molecular weight heparin s.c., methylprednisolone i.v.)	70 received a single i.v. dose of 400 mg while 91 did not	NA	Hospitalized, 2 (2.9%) requiring mechanical ventilation	Mortality rate
Guaraldi et al., 2020 [34]	Italy	544 patients (179 cases and 365 controls)	Laboratory-confirmed cases	64 [range 54–72], younger than controls (*p* = 0.0064)	127 (71%); comparable in terms of gender	Tocilizumab + steroids, hydroxychloroquine, azithromycin, antivirals and antiretrovirals, such as darunavir–cobicistat or lopinavir/ritonavir, anticoagulants	8 mg/kg i.v. up to a maximum of 800 mg administered twice, 12 h apart; 162 mg administered s.c. in two simultaneous doses; *n* = 91 s.c., *n* = 88, i.v.	At the time of hospital admission	Hospitalized patients	Survival rate Need for mechanical ventilation
Holt et al., 2020 [35]	USA	62 patients (32 cases and 30 controls)	Laboratory-confirmed cases	NA	NA	Tocilizumab + standard care (NA)	NA	NA	Hospitalized patients	Mortality
Ip et al., 2020 [36]	USA	547 patients (134 cases and 413 controls)	Laboratory-confirmed cases	62 [range 53–70], younger than controls (*p* < 0.0001)	99 (73.9%), less females among cases than among controls	Tocilizumab + steroids, hydroxychloroquine, azithromycin	104 (78%) receiving 400 mg (96%), followed by 800 mg (1%), 8 mg/kg (1%), 4 mg/kg (1%), and missing dosing (1%)	After entering the ICU	All admitted to the ICU (29 admitted on first day to the ICU)	Survival rate
Kewan et al., 2020 [37]	USA	51 patients (28 cases and 23 controls)	Laboratory-confirmed cases	62 [range 53–71], younger than controls	20 (71%), less females among cases than among controls	Tocilizumab + standard care (azythromicin, hydroxychloroquine, steroids)	8 mg/kg up to 400 mg as a 60 min single i.v. infusion	Following admission based on clinical parameters	Hospitalized/admitted to the CU	Mortality rate Length of stay ICU admission Need for mechanical ventilation Bacterial infection
Kimmig et al., 2020 [38]	USA	60 patients (28 cases versus 32 controls)	Laboratory-confirmed cases	63.86 ± 16.04	20 (71.4%), less females among cases than males among controls	Tocilizumab + standard care (NA)	400–800 mg (*n* = 3 patients received a second dose; *n* = 1 patient received a single dose of 800 mg)	NA	All admitted to the ICU	Mortality rate Infection rate
Klopfenstein et al., 2020 [39]	France	45 patients (20 cases versus 25 controls), with severe disease	Laboratory-confirmed cases and clinical suspicion, exclusion of patients receiving non standard care treatment (such as IVIG), exclusion of patients with moderate disease	76.8 ± 11 [range 52–93]; no differences with controls	NA	Tocilizimab + standard care (hydroxychloroquine, lopinavir-ritonavir, antibiotics, and corticosteroids)	At least 1 or 2 doses	13 days after symptoms onset, 7 days after admission/hospitalization	Hospitalized, none admitted to the ICU	ICU admission and death (composite clinical outcome)
Martínez-Sanz et al., 2020 [40]	Spain	1229 patients (260 cases and 969 controls)	Laboratory-confirmed cases (nasopharyngeal swabs or other valid respiratory samples)	65 [range 55–76] (younger than controls, *p* = 0.017)	*n* = 191 (73%); less females among cases than among controls (*p* < 0.001)	Tocilizumab + standard care (steroids, hydroxychloroquine, azithromycin, lopinavir/ritonavir)	600 mg (IQR 600–800 mg)	After 4 (IQR 3–5) days since admission	Hospitalized, 50 (19%) admitted to the ICU	ICU admission Death rate
Mikulska et al., 2020 [41]	Italy	196 patients (85 cases and 111 controls)	Laboratory-confirmed cases	32–85	59	Tocilizumab + standard care (hydroxychloroquine, darunavir/ritonavir, methylprednisolone)	8 mg/kg i.v. or 162 mg s.c.	After 3 days from admission	Hospitalized	Mortality rate
Moreno-Garcia et al., 2020 [42]	Spain	171 patients (77 cases versus 94 controls)	Laboratory-confirmed cases (*n* = 68, 88.3%)	61.5 ± 12.4	53 (68.8%)	Tocilizumab + standard care (lopinavir/ritonavir plus hydroxychloroquine, azithromycin, steroids such as methylprednisolone, heparin)	400–600 mg i.v.	After admission based on clinical course	8 (10.3%) admitted to the ICU	Oxygen therapy
Moreno-Pérez et al., 2020 [43]	Spain	236 patients (77 cases and 159 controls)	Laboratory-confirmed cases	62.0 [range 53.0–72.0], slightly older than controls but borderline significant	64.9 (50/77)	Tocilizumab + standard care (hydroxychloroquine, lopinavir/ritonavir, and azithromycin)	Initial dose of 600 mg, with a second or third dose (400 mg) in case of persistent or progressive disease	2.0 days [range 1.0–4.0] after admission	Hospitalized, forty-two patients (54.5%) admitted to the ICU	Mortality
Narain et al., 2020 [44]	USA	3098 patients (364 cases and 2734 controls)	Laboratory confirmed cases	64.91, comparable with controls	267/364, less females among cases than among controls	Tocilizumab + standard care (hydroxychloroquine, remdesivir, ritonavir/lopinavir, steroids)	NA	52.66 h after admission (approximately 2 days after admission)	Hospitalized	Mortality
Omrani et al., 2020 [45]	Qatar	1409 patients (111 cases and 1298 controls)	Laboratory confirmed cases	NA	NA	Tocilizumab + standard care (hydroxychloroquine, antibiotics, ribavirin, interferon, lopinavir/ritonavir)	NA	NA	Hospitalized	ICU admission
Pandolfi et al., 2020 [46]	Italy	28 patients (8 cases and 20 controls)	Laboratory confirmed cases	NA	NA	Tocilizumab + standard care (hydroxychloroquine, remdesivir, ritonavir/lopinavir, steroids)	NA	NA	Admitted to the ICU	Mortality
Patel et al., 2020a [47]	USA	129 patients (24 cases and 105 controls)	Laboratory confirmed cases	NA	NA	Tocilizumab + standard care (Azithromycin, hydroxychloroquine and steroids)	NA	NA	NA	Progression to invasive ventilation
Patel et al., 2020b [48]	USA	104 patients (6 cases and 98 controls)	Laboratory confirmed cases	NA	NA	Tocilizumab + standard care (remdesivir, steroids, hydroxychloroquine antibiotics)	NA	NA	NA	Need of invasive ventilation
Pérez-Tanoira et al., 2020 [49]	Spain	382 patients (36 cases and 346 controls)	Laboratory confirmed cases	NA	NA	Tocilizumab + standard care (darunavir/cobicistat, lopinavir/ritonavir, chloroquine/hydroxychloroquine, interferon β-1B, antibiotics)	NA	NA	NA	Mortality rate
Perrone et al., 2020 [50]	Italy	1158 patients (708 cases, 180 from ITT phase 2 trial and 528 from ITT validation trial, and 450 controls)	Laboratory confirmed cases	NA, younger than controls (*p* = 0.04)	82.8% and 79.5%, less females among cases than among controls	Tocilizumab + standard care: antivirals/antiretroviral, Lopinavir/Ritonavir, Remdesivir, (hydroxy)chloroquine, colchicine, immune suppressor, antibiotics, azythromicin, ceftriaxone, linezolid, steroids, heparin	400–800 mg	138 (76.7%) and 404 (76.5%) within 3 days from registration	Admitted to the ICU (NA)	Mortality rate
Potere et al., 2020 [51]	Italy	80 patients (40 cases and 40 controls)	Laboratory-confirmed cases	56.0 [range 50.3–73.2]; age-matched with controls	26 (65.0%); gender-matched with control	Tocilizumab + standard care	324 mg, given as two concomitant subcutaneous injection	NA	NA	Infection Mortality rate Need for mechanical ventilation and/or death
Quartuccio et al., 2020 [52]	Italy	111 consecutive patients (42 cases versus 69 controls)	Laboratory-confirmed cases	58.5 ± 13.6, older than controls	77 (69.4%)	Tocilizumab + standard care (antiviral therapy, anticoagulants, hydroxychloroquine, antibiotics and glucocorticoids)	8 mg/kg i.v. as a single infusion; 2 patients received 200 mg/day s.c. for 3 consecutive days	8.4 ± 3.7 days after symptoms onset	27 transferred to ICU (3 before receiving Tocilizumab and 24 after hospital admission); 26 intubated and 1 with non invasive ventilation	Mortality rate
Ramaswamy et al., 2020 [53]	USA	86 patients (21 cases versus 65 controls)	Laboratory-confirmed cases	63.2 ± 15.6, age-matched	13 (61.9%), gender-matched	Tocilizumab + standard care: azithromycin, hydroxychloroquine, corticosteroids	400–800 mg; seven receiving a single dose of 800 mg	10 prior to mechanical ventilation, 11 after mechanical ventilation	Admitted to the ICU (*n*= 10, 47.6%), with 13 requiring mechanical ventilation (61.9%)	Mortality rate
Rodríguez Molinero et al., 2020 [54]	Spain	418 consecutive patients (96 cases and 322 controls)	Laboratory-confirmed cases	NA, age-matched sub-analysis	NA, gender-matched sub-analysis	Tocilizumab + standard care (hydroxychloroquine, lopinavir/ritonavir, azithromycin, steroids such as metilpredinosolone)	An initial dose of 600 mg i.v., a second dose of 400–600 mg at 12 h, and a third optional dose of 400 mg	NA	Hospitalized patients	Mortality rate Time to discharge
Rojas-Marte et al., 2020 [56]	USA	193 patients (96 cases versus 97 controls)	Laboratory-confirmed cases	58.8 ± 13.6, age-matched	*n* = 74 (77.1%), gender-marched	Tocilizumab + standard care (hydroxychloroquine, azithromycin, steroids, anticoagulants, remdesivir, vitamin C, zinc, antibiotics for suspected bacterial infections)	NA	NA	Hospitalized patients, 61 (63.5%) requiring invasive ventilation	Mortality rate
Rossi et al., 2020 [56]	France	246 patients (106 cases versus 140 controls)	Laboratory-confirmed cases	64.3 ± 13, younger than controls	66%, less females among cases than among controls	Tocilizumab + standard care (antibiotics, betalactamin, macrolides, antivirals, hydroxychloroquine, lopinavir/ritonavir, immunosuppressants and/or corticosteroids, Baricitinib)	A single dose of 8 mg/kg (400 mg)	Within 1 ± 1 day after hospitalization	Hospitalized patients	Mortality rate and all-cause mortality rate Need for mechanical ventilation
Roumier et al., 2020 [57]	France	59 patients (30 cases versus 29 controls) with severe disease	Laboratory-confirmed cases (*n* = 29)	Mean 58.8 ± 12.4; median 50; younger than controls (*p* = 0.001)	*n* = 24 80%	Tocilizumab + standard care (hydroxychloroquine and azithromycin); 2 controls received steroids	8 mg/kg i.v.	14.1 ± 3.5 days after symptoms onset	Admitted to the ICU (*n* = 7, 23%), 10 under invasive ventilation (33.3%)	Need for mechanical ventilation Mortality rate ICU admission
Sisó-Almirall et al., 2020 [58]	Spain	322 patients (27 cases and 295 controls)	Laboratory-confirmed cases	NA	NA	Tocilizumab + standard care (remdesivir, hydroxychloroquine, steroids, anti-coagulation, antibiotics)	NA	NA	Hospitalized, with some admitted to the ICU	Mortality and/or ICU admission
Somers et al., 2020 [59]	USA	154 patients (78 cases and 76 controls) with severe disease, requiring mechanical ventilation	Laboratory-confirmed cases	55 ± 14.9, younger than controls (*p* = 0.05)	*n* = 53, 68%	Tocilizumab + standard care (remdesivir, hydroxychloroquine, steroids, anti-coagulation)	Single dose of 8 mg/kg up to a maximum of 800 mg	24 h prior to intubation, in 26% patients > 48 h after intubation	All on mechanical ventilation; 40 transferred on mechanical ventilation; all hospitalized, no one admitted to the ICU	Mortality rate
Wadud et al., 2020 [60]	USA	94 patients (44 cases versus 50 controls)	Laboratory-confirmed cases	55.5	NA	Tocilizumab + standard care (hydroxychloroquine, azithromycin, steroids-hydrocortisone/methylprednisolone/dexamethasone))	NA	NA	Admitted to the ICU (not specified how many subjects); all requiring mechanical ventilation	Mortality rate

**Table 3:** Main findings and quality assessment of included studies.

Reference	Main Findings	Adjustment of the Outcome(s)	Effects of Tocilizumab on Clinical and Lab Parameters	Publication Status	Quality Appraisal
Allenbach et al., 2020 [22]	2 (2.1%) ICU-free and/or alive versus 4 (8.7%) ICU-admitted and/or died (*p* = 0.087) among those receiving tocilizumab	No	NA	Pre-print	Selection = 2, comparability = 0, exposure = 2
Ayerbe et al., 2020 [23]	89/421 (21.14%) versus 197/1598 (12.33%)	No	NA	Peer-reviewed	Selection = 2, comparability = 0, exposure = 2
Campochiaro et al., 2020 [24]	5/32 deaths; mortality rate among cases (16%) and among controls (*n* = 11/33, 33%) was not different (*p* = 0.150) for the first outcome 4/32 (13%) versus 2/33 (6%) (*p* = 0.43) for the second outcome	No	Age predicted survival and PaO_2_/FiO_2_ ratio predicted clinical improvement	Peer-reviewed	Selection = 2, comparability = 2 (no difference with controls in terms of co-morbidities and respiratory parameters), exposure = 3
Capra et al., 2020 [25]	Overall, 2/62 (3.22%) versus 11/23 (47.8%) with HR 0.035 ([95%CI 0.004–0.347], *p* = 0.004) Among those with a concluded outcome 2/25 (8%) versus 11/19 (57.9%)	Age, co-morbidities and PCR baseline levels	None (no changes in procalcitonin levels)	Peer-reviewed	Selection = 2, comparability = 2, exposure = 3
Carvalho et al., 2020 [26]	17.2% (5/29) versus 16.7% (4/24); adjusted OR 3.97 ([95%CI 0.28–57.2], *p* = 0.3) for the first outcome 11 (38%) versus 4 (17%); adjusted OR 1.73 ([95%CI 0.22–13.82], *p* = 0.6) for the second outcome	Yes (multivariate analysis) Yes (multivariate analysis)	None Mechanical ventilation (*p* = 0.006)	Pre-print	Selection = 1, comparability = 1 (use of steroids 83% versus 37%, *p* = 0.001; differences in terms of biochemical parameters), exposure = 3
Colaneri et al., 2020 [27]	3/21 versus 12/91, no significant effect, with OR 0.11 ([95%CI 0.00–3.38], *p* = 0.22)	Yes (nearest neighbor propensity score matching)	None	Peer-reviewed	Selection = 2, comparability = 2, exposure = 3
	5/21 versus 19/91 with OR 0.78 ([95%CI 0.06–9.34], *p* = 0.84)	Yes			
Crotty et al., 2020 [28]	20% versus 4.9%, (*p* = 0.013)	No	NA	Pre-print	Selection = 2, comparability = 0, exposure = 2
de la Rica et al., 2020 [29]	10/11 versus 11/47	No	NA	Pre-print	Selection = 2, comparability = 0, exposure = 2
Edwards and McGrail, 2020 [30]	2/11 versus 6/24	No	NA	Pre-print	Selection = 2, comparability = 0, exposure = 2
Fernández-Cruz et al., 2020 [31]	24/180 versus 47/283	No	NA	Peer-reviewed	Selection = 2, comparability = 0, exposure = 2
Garibaldi et al., 2020 [32]	6/39 among cases versus 107/793 among controls	No	NA	Pre-print	Selection = 2, comparability = 0, exposure = 2
Gokhale et al., 2020 [33]	33 (47.1%) versus 61 (67%) (*p* = 0.011)	No	NA	Peer-reviewed	Selection = 2, comparability = 0, exposure = 3
Guaraldi et al., 2020 [34]	13 (7%) versus 73 (20%), *p* = 0.0007; unadjusted HR 0.60 ([95%CI 0.43–0.84], *p* = 0.0030) 33 (18%) among cases versus 57 (16%) among controls	Yes (adjusted; also, inverse probability weighting model is presented)	NA	Peer-reviewed	Selection = 2, comparability = 2, (not comparable in terms of co-morbidities, clinical, biochemical and respiratory parameter) exposure = 3
Holt et al., 2020 [35]	10/32 versus 9/30	Yes (matched)	None	Pre-print	Selection = 2, comparability = 2, exposure = 3
Ip et al., 2020 [36]	Adjusted HR 0.76 ([95%CI 0.57–1.00], *p* = 0.053), 46% versus 56%	Yes (propensity-score model)	None	Pre-print	Selection = 2, comparability = 2 (differences in terms of co-morbidities, respiratory parameters and use o antibiotics and hydroxychloroquine), exposure = 3
Kewan et al., 2020 [37]	3 (11) 2 (9)	No	NA	Peer-reviewed	Selection = 2, comparability = 0, exposure = 3
	11 [6–22.25] versus 7 [5–13.5]				
	86% versus 70%, *p* = 0.19				
	75% versus 48%, *p* = 0.046				
	Five (18%) versus five (22%) (*p* = 0.74)				
Kimmig et al., 2020 [38]	12 (42.9%) versus 8 (25%)	No	None	Pre-print	Selection = 2, comparability = 1, exposure = 3
	18/28 versus 10/32				
Klopfenstein et al., 2020 [39]	25% (cases; *n* = 5/20) versus 72% (controls; *n* = 18/25) overall (*p* = 0.002); ICU admission *n* = 0 versus *n* = 11 (*p* < 0.0001); deaths *n* = 5 versus *n* = 12 (*p* = 0.066), mechanical ventilation *n* = 0 versus *n* = 8 (*p* = 0.006), hospitalization *n* = 3 versus *n* = 2 (*p* = 0.642), discharge *n* = 11 versus *n* = 11 (*p* = 0.463).	No	NA	Peer-reviewed	Selection = 2, comparability = 1 (differences in terms of biochemical and respiratory parameters), exposure = 3
Martínez-Sanz et al., 2020 [40]	50 (19%) among cases versus 32 (3%) among controls (*p* < 0.001)	Yes, using inverse probability treatment weighting	High CRP values	Pre-print	Selection = 2, comparability = 2 (non comparable in terms of co-morbidities, respiratory and laboratory parameters), exposure = 3
	61 (23%) among cases versus 120 (12%) among controls (*p* < 0.001); unadjusted HR 1.53, ([95%CI 1.20–1.96], *p* = 0.001), adjusted HR 0.34 ([95%CI 0.16–0.72], *p* = 0.005), stratifying according to CRP levels; composite index adjusted HR 0.39 ([95%CI 0.19–0.80, *p* = 0.011) stratifying according to CRP levels				
Mikulska et al., 2020 [41]	9/85 versus 36/111	No (adjustment is done but not for this specific outcome)	NA	Pre-print	Selection = 2, comparability = 0, exposure = 3
Moreno-Garcia et al., 2020 [42]	ICU admission (10.3% versus 27.6%, *p* = 0.005) Need of invasive ventilation (0% versus 13.8%, *p* = 0.001); ICU admission and/or death composite outcome OR 0.03 ([95%CI 0.007–0.1], *p* = 0.0001)	Yes	Co-morbidities (hypertension, heart diseases and lymphoma), need of oxygen at day 1, CRP > 16 mg/dL and cardiovascular, renal or respiratory (ARDS, invasive ventilation) complications predicted ICU admission and/or death	Pre-prrint	Selection = 1, comparability = 1 (age-, gender-matched, differences in use f steroids) exposure = 2
Moreno-Pérez et al., 2020 [43]	10/77 versus 3/159	No	None	Peer-reviewed	Selection = 2, comparability = 1 (differences in terms of clinical, biochemical and respiratory parameters), exposure = 3
Narain et al., 2020 [44]	Adjusted HR 0.718 ([95%CI 0.403–1.280, *p* = 0.2615) for tocilizumab only, adjusted HR 0.459 ([95%CI 0.399–0.622), *p* < 0.0001) for tocilizumab plus steroids	Yes	None	Pre-print	Selection = 2, comparability = 2, exposure = 3
Omrani et al., 2020 [45]	99/111 versus 12/1298	No	NA	Pre-print	Selection = 2, comparability = 0, exposure = 2
Pandolfi et al., 2020 [46]	4/8 versus 8/20	No	NA	Pre-print	Selection = 2, comparability = 0, exposure = 2
Patel et al., 2020a [47]	14 (15.7%) versus 10 (25.0%) (*p* = 0.211)	No	NA	Pre-print	Selection = 2, comparability = 0, exposure = 2
Patel et al., 2020b [48]	5 (13.89%) versus 1 (1.52%) (*p* = 0.011)	No	NA	Pre-print	Selection = 2, comparability = 0, exposure = 2
Pérez-Tanoira et al., 2020 [49]	10/36 versus 95/346	No	NA	Pre-print	Selection = 2, comparability = 0, exposure = 2
Perrone et al., 2020 [50]	67 and 158 overall deaths; 36/180 versus 31/119; 99/495 versus 59/331	No	Older age and low PaO_2_/FiO_2_ ratio predicted mortality rate	Pre-print	Selection = 2, comparability = 0 (differences in terms of respiratory parameters), exposure = 2
Potere et al., 2020 [51]	1 (2.5%) among cases developed bacterial pneumonia versus 3 (7.5%) among controls	No	NA	Peer-reviewed	Selection = 2, comparability = 2, exposure = 3
	2 (5%) versus 11 (27.5% (*p* = 0.006)				
	IMV or death (2 (5%) vs 12 (30%), *p* = 0.003				
Quartuccio et al., 2020 [52]	9.5% among cases versus 0.0% among controls	No	Co-morbidities and superinfections	Peer-reviewed	Selection = 2, comparability = 0 (differences in terms of use of drugs, clinical and biochemical parameters)), exposure = 3
Ramaswamy et al., 2020 [53]	3/21 deaths versus 8/65 deaths, HR 0.25 [95%CI 0.07–0.90], RR 0.472 [95%CI 0.449–0.497]	Yes	Being treated with tocilizumab and age at admission predicted survival rate	Pre-print	Selection = 2, comparability = 2, exposure = 3
Rodríguez Molinero et al., 2020 [54]	Adjusted OR 0.99 ([95%CI 0.30–3.27], *p* = 0.990)	Yes (brute-force matching algorithm refined by propensity score)	None	Pre-print	Selection = 2, comparability = 2, exposure = 3
	*p* = 0.472				
Rojas-Marte et al., 2020 [56]	43 (44.8%) deaths versus 55 (56.7%) (*p* = 0.09); excluding intubated patients, 2 (6.1%) versus 9 (26.5%) (*p* = 0.024)	No	NA	Pre-print	Selection = 2, comparability = 1, exposure = 3
Rossi et al., 2020 [56]	Adjusted HR 0.34 ([95%CI 0.22–0.52], *p* < 0.0001); adjusted HR 0.29 ([95%CI 0.17–0.53], *p* < 0.0001)/ HR 0.42 ([95%CI 0.22–0.82], *p* = 0.008)	Yes	SpO_2_/FiO_2_ ratio and CKD predicted mortality rate	Pre-print	Selection = 2, comparability = 2 (differences in terms of use of antibiotics, respiratory parameters), exposure = 3
	HR 0.49 ([95%CI 0.30–0.81], *p* = 0.00)				
Roumier et al., 2020 [57]	OR 0.42 ([95%CI 0.20–0.89], *p* = 0.025)	Yes (age, gender, disease severity)	NA	Pre-print	Selection = 1, comparability = 2 (differences in terms of co-morbidities), exposure = 3
	3 versus 9 deaths (*p* = 0.041), 4 discharged from the ICU and 6 from hospital; at the univariate analysis, OR 0.25 ([95%CI 0.05–0.95], *p* = 0.04); at the multivariate analysis, no statistical significance; considering those treated outside the ICU tocilizumab resulted protective	Yes (age, gender, disease severity)			
	OR 0.17 ([95%CI 0.06–0.48], *p* = 0.001)	Yes (age, gender, disease severity)	None		
Sisó-Almirall et al., 2020 [58]	Adjusted OR 3.17 ([95%CI 1.22–7.88], *p* = 0.013)	Yes (multivariate analysis)	None	Pre-print	Selection = 2, comparability = 2, exposure = 3
Somers et al., 2020 [59]	14 (18%) versus 27 (36%), *p* = 0.01; *p* = 0.0189 at the Kaplan–Meyer analysis; adjusted HR 0.55 [95%CI 0.33–0.90]; when stratifying into patients with super-infections, no difference in 28-day case fatality rate (22% versus 15%, *p* = 0.42)	Yes (propensity score-based inverse probability treatment weighting)	None	Peer-reviewed	Selection = 2, comparability = 2 (differences n terms of clinical, respiratory and biochemical parameters), exposure = 3
Wadud et al., 2020 [60]	61.36 % versus 48 % in the control group (17 deaths versus 26)	Yes (cases and controls matched in terms of age, sex, BMI and HS score- calculated using inflammatory markers- ferritin, triglycerides, AST and fibrinogen)	NA	Pre-print	Selection = 2, comparability = 2, exposure = 3

## Data Availability

All data relevant to the study are included in the article.
